# Genetic variation in the myeloperoxidase gene and cognitive impairment in Multiple Sclerosis

**DOI:** 10.1186/1477-5751-5-3

**Published:** 2006-02-27

**Authors:** I Manna, P Valentino, A La Russa, F Condino, R Nisticò, M Liguori, A Clodomiro, V Andreoli, D Pirritano, R Cittadella, A Quattrone

**Affiliations:** 1 Institute of Neurological Science, National Research Council, Cosenza, Italy; 2 Department of Medical Sciences, Institute of Neurology, University "Magna Graecia", Catanzaro, Italy

## Abstract

There is evidence that multiple sclerosis (MS) may associated with cognitive impairment in 25 to 40% of cases. The gene encoding myeloperoxidase (MPO) is involved in molecular pathways leading to β-amyloid deposition. We investigated a functional biallelic (G/A) polymorphism in the promoter region (-463) of the MPO gene in 465 patients affected by MS, divided into 204 cognitively normal and 261 impaired. We did not find significant differences in allele or genotype distributions between impaired and preserved MS patients. Our findings suggest that MPO polymorphism is not a risk factor for cognitive impairment in MS.

## Findings

Multiple sclerosis (MS) is a chronic inflammatory disease of the central nervous system (CNS), characterised by primary demyelination with relative axonal sparing and by a clinical course that varies from relapsing remitting (RR) to chronic progressive (CP). Cognitive dysfunction occurs in 25–40% of patients with MS, and it is often a major cause of disability in patients with the disease [1]. Although the pathogenesis of MS is not fully understood the role of genetic factors is firmly established [2]. Such a genetic factor might be better identified through association studies which look for an increased frequency of a particular genetic marker or allele among the affected individuals as compared to unaffected individuals. The myeloperoxidase (MPO) gene encodes for an enzyme that catalyses a reaction between hydrogen peroxide and chloride to generate hypochlorous acid, a potent oxidant leading to oxidising conditions that are known to increase β-amiloid protein (Aβ) deposition [3]. The MPO gene, at 17q23.1, contains a functionally important G to A base substitution polymorphism, 463 bases upstream from the transcription start site, which has been found to cause a decreased transcriptional activity in cellular transfection assays due to the destruction of an SP1 binding site [4]. A few studies have investigated the -463 G/A MPO promoter polymorphism in MS. Nagra and co-workers [5] found an overrepresentation of the GG genotype in women with early onset MS. Two subsequent studies found no association between the MPO promoter polymorphism and MS [6,7]. In a recent work Zakrewska-Pniewska and co-workers analysed the relationship between APOE and MPO genes' polymorphisms and MS, and they found that the genotype GG of MPO was related to more pronounced brain atrophy [8]. Owing to the protein product of the MPO gene being involved in AD pathology, possibly through oxidation of Aβ or ApoE, promoting their aggregation into insoluble complexes, or directly through oxidation-induced damage to associated neurons [9], there is biologic evidence implicating MPO in the cognitive decline in patients with MS, but no definite data are currently available on the possible role of MPO polymorphism in the development of cognitive decline in MS. This study was mainly designed to investigate whether some clinical and individual variables and the occurrence of the -463 G/A promoter polymorphism of the MPO gene may be associated with cognitive impairment in patients with MS. The sample included in this study consisted of 465 patients affected by MS data on our patients were reported in greater detail elsewhere [10]. Informed consent to perform molecular genetic studies was obtained from all patients.

Genomic DNA was prepared from leukocytes harvested from whole blood using standard methods. The PCR-RFLP based assay was used to characterise the wild-type (G) and variant (A) MPO alleles at position -463 [11]. Statistical analyses were performed with Statistical Package for Social Sciences software SPSS (version 12.0, Chicago, IL, USA) for Windows '98/'00. In our sample of consecutive patients affected by MS, we found the cognitive deterioration was present at different degrees of severity in the majority of the patients. More in detail, two-hundred and four patients (44%) were found to be cognitively preserved by the neuropsychological evaluation, whereas 261 (56%) failed at least one test and were therefore considered cognitively impaired. The cognitively impaired group differed from the preserved group in the following characteristics: a longer disease duration, a high EDSS score, a greater proportion of individuals with secondary progressive form and a lower education. No difference was found in the number of failed neuropsychological test among subjects with different polymorphic variants (p = 0.805; Kruskal-Wallis test). No significant difference was found in the genotypic (p = 0.649; Pearson χ^2^-test) or allelic distribution (p = 0.517; Pearson χ^2^-test) of the -463 G/A promoter polymorphism of the MPO gene between preserved and impaired subjects (Table). Furthermore, considering a power of 80% and a significance level of 0.05, the power calculation for the A allele shows that the ORs detectable as significant resulted lower than 0.618 and higher than 1.527. These results suggest that the -463 G/A promoter polymorphism of the MPO gene does not confer a risk of cognitive impairment in patients with MS.

## List of abbreviations

CI confidence interval

## Authors' contributions

IM, PV, A La R, partecipated in the study design, carried out the data collection and analysed the results. FC performed the statistical analysis. RN, ML, AC, VA, DP and RC partecipated to acquisition of data. AQ conceived the study and partecipated in its design and coordination and drafted the manuscript.

**Table 1:** Genotypes and allele frequencies of the MPO polymorphism

	Preserved N = 204	Impaired N = 261	p-value	OR*	95% CI*
Genotype frequency					
AA	12 (5.9%)	16 (6.1%)	0.649	0.956	0.435–2.102
AG	70 (34.3%)	79 (30.3%)		0.852	0.569–1.273
GG	122 (59.8%)	166 (63.6%)		1.0	
Allele frequency					
A	94 (23.0%)	111 (21.3%)	0.517	0.909	0.665–1.244
G	314 (77.0%)	411 (78.7%)		1.0	

*Odds Ratios and CI were estimated using logistic regression adjusted for age and sex.
